# Spin-charge separation and quantum spin Hall effect of $\beta$-bismuthene

**DOI:** 10.1038/s41598-023-38491-1

**Published:** 2023-07-14

**Authors:** Alexander C. Tyner, Pallab Goswami

**Affiliations:** 1 Graduate Program in Applied Physics, Northwestern University, Evanston, IL 60208 USA; 2 Department of Physics and Astronomy, Northwestern University, Evanston, IL 60208 USA

**Keywords:** Topological matter, Topological insulators

## Abstract

Multiple works suggest the possibility of classification of quantum spin Hall effect with magnetic flux tubes, which cause separation of spin and charge degrees of freedom and pumping of spin or Kramers-pair. However, the *proof of principle* demonstration of spin-charge separation is yet to be accomplished for realistic, ab initio band structures of spin-orbit-coupled materials, lacking spin-conservation law. In this work, we perform thought experiments with magnetic flux tubes on $\beta$-bismuthene, and demonstrate spin-charge separation, and quantized pumping of spin for three insulating states, that can be accessed by tuning filling fractions. With a combined analysis of momentum-space topology and real-space response, we identify important role of bands supporting even integer invariants, which cannot be addressed with symmetry-based indicators. Our work sets a new standard for the computational diagnosis of two-dimensional, quantum spin-Hall materials by going beyond the $\mathbb {Z}_{2}$ paradigm and providing an avenue for precise determination of the bulk invariant through computation of quantized, real-space response.

## Introduction

Chern insulators^1–3^ and quantum spin Hall (QSH) insulators^4–9^ are two prominent examples of two-dimensional (2D) topological phases of matter. For clean, crystalline materials, supporting momentum-conservation law, they respectively arise due to the existence of net Chern number ($\mathfrak {C}_{GS} \in \mathbb {Z}$)^2,3,10^, and net relative or spin Chern number ($\mathfrak {C}_{R,GS} \in \mathbb {Z}$)^4,5,11–15^ for completely occupied bands. The Chern number $\mathfrak {C}_{GS}$ of time-reversal-symmetry ($\mathcal {T}$) breaking materials describes quantized Abelian Berry flux ($2 \pi \mathfrak {C}_{GS}$) through 2D Brillouin zone (BZ), which can be calculated using TKNNY formula^2^. In the presence of electronic interactions and impurities, the Chern number can be determined by imposing twisted boundary conditions (TBC) in real space^10^. Above all, the quantized topological response can be directly probed by following Laughlin’s thought experiment with magnetic flux tube^1^, which binds electric charge $e \;\mathfrak {C}_{GS} \; \phi /\phi _0$. When $\phi$ is adiabatically tuned from 0 to $\phi _0=h/e$, one observes quantized pumping of electric charge $\delta Q = e \; \mathfrak {C}_{GS}$.

In contrast to $\mathfrak {C}_{GS}$, $\mathfrak {C}_{R,GS}$ describes quantized, non-Abelian Berry flux $2 \pi \mathfrak {C}_{R,GS}$ through 2D BZ. The definition of non-Abelian Berry flux for spin-orbit-coupled materials has many subtleties due to the absence of continuous spin-rotation symmetry or spin-conservation law. Thus, Kane and Mele formulated $\mathbb {Z}_2$-classification of QSH effect of $\mathcal {T}$-preserving systems, which distinguishes between odd and even integer values of $\mathfrak {C}_{R,GS}$^4^. For generic $\mathcal {T}$-preserving systems, the $\mathbb {Z}_2$ invariant $(-1)^{\mathfrak {C}_{R,GS}}$ can be calculated from the gauge-invariant spectrum of Wilson loops (Wannier charge centers) of non-Abelian Berry connection, defined in momentum space^16–19^. Furthermore, for materials preserving space-inversion ($\mathcal {P}$) and $\mathcal {T}$ symmetries, it can be identified from a symmetry-based indicator, which is the product of parity eigenvalues at time-reversal-invariant momentum points^8^.

To go beyond the $\mathbb {Z}_2$-classification of QSH insulators provided by the Fu-Kane strong index, without relying on spin and momentum conservation laws, various authors have considered the role of generalized TBCs and generalization of Laughlin’s thought experiment^11,12,20^. Since QSH insulators are expected to show cross-correlated response between spin and charge channels, one employs mixed TBCs: say $e^{i \theta _x \sigma _0}$ along *x* direction and $e^{i \theta _y \sigma _3}$ along *y* direction, and $\theta _{x}$, $\theta _y$ are varied between 0 and $2 \pi$^11^, where Pauli matrix $\sigma _3$ acts on spin index. While this theoretical framework allows identification of signed spin Chern numbers, the implementation of spin-dependent TBC requires detailed understanding of underlying basis states or the choice of spin-quantization axis, and how the spin-conservation law is violated. Hence, their use for realistic numerical tight-binding models has practical limitations.

**Figure 1:**
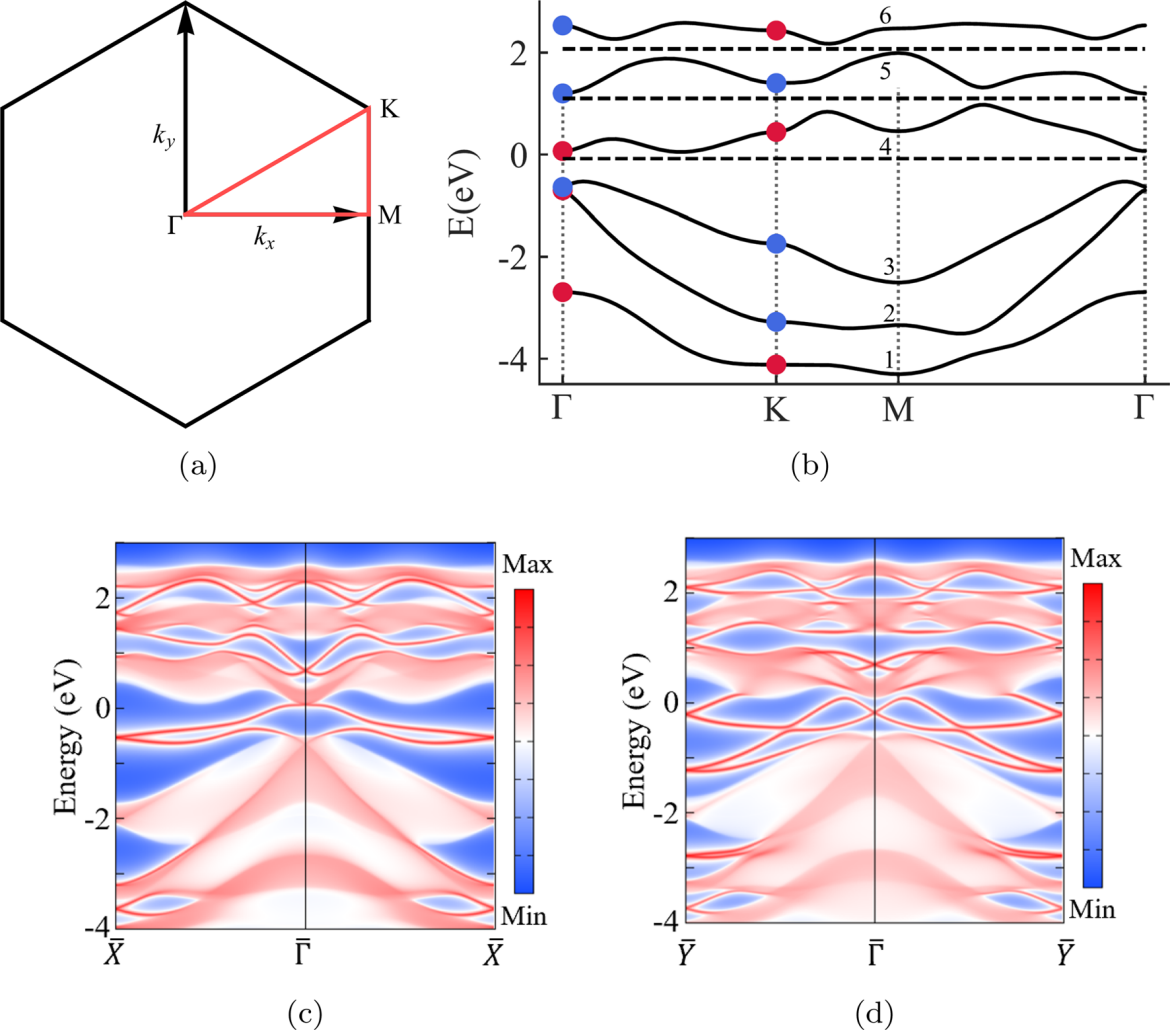
(**a**) Hexagonal Brillouin zone of $\beta$-bismuthene. (**b**) Band structure along high-symmetry path $\Gamma -M-K-\Gamma$ and energies are measured with respect to a reference value $E_0=0$. Bands are numbered according to their energies at $\Gamma$ point, such that $E_{n}(\Gamma )<E_{n+1}(\Gamma )$. Parity eigenvalues $\pm 1$ at time-reversal-invariant-momentum points are denoted by red and blue dots, respectively. The dashed lines correspond to three representative values of Fermi energy, tuned in direct band gaps, leading to 1/2-, 2/3-, and 5/6-filled insulators. They support non-trivial $\mathbb {Z}_2$-invariant $\nu _{0,GS}=1$. (c,d) Spectral density on the (**c**) armchair and (**d**) zigzag surfaces under open boundary conditions. The mid-gap edge-modes, connecting bulk valence and conduction bands imply first-order topology of insulating states.

Such difficulties can be circumvented by following the proposal of Qi and Zhang^21^, and Ran et al.^22^ to diagnose QSH states with magnetic flux tubes (i.e., gauging of conserved number currents). Employing 4-band models of QSH states with $|\mathfrak {C}_{R,GS}|= 1$, they have shown that a flux tube, carrying half of flux quantum $\phi = \frac{\phi _0}{2}$ ($\mathcal {T}$-invariant, $\pi$ flux) binds two degenerate, mid-gap states. At half-filling, one of these modes is occupied, and the ground state exhibits 2-fold-degeneracy (*SU*(2)-doublet). Consequently, the flux tube remains charge-neutral, and carries spin quantum number $\pm \frac{1}{2}$. When both modes are occupied (empty), the flux tube carries electric charge $-e$ ($+e$), and spin quantum number 0 (*SU*(2)-singlets). Such states can be accessed by doping insulators with one electron (hole). This solitonic mechanism of *spin-charge separation* (SCS) is similar to what is known for polyacetelene^23,24^ and topologically ordered, correlated systems^25^. When $\mathcal {T}$ is broken by generic values of $\phi$, the bound modes and the half-filled ground state become non-degenerate. But the flux tube continues to bind spin and no electric charge. By adiabatically tuning $\phi$ from 0 to $\phi _0$, quantized pumping of spin (one Kramers-pair) can be observed [see supplementary material].

**Figure 2:**
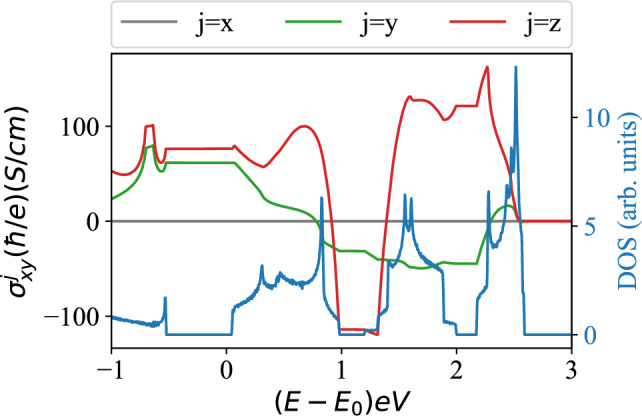
Spin Hall conductivity $\sigma ^{j}_{xy}$ of $\beta$-bismuthene from first principles calculations, as a function of energy. Three insulating states can be identified from direct gaps in density of states. In addition to showing plateau-like features, $\sigma ^{z}_{xy}$ shows sharp change of sign, when bands 4 and 5 become occupied. While these results cannot capture precise topological properties, they indicate topological non-triviality of these $\mathbb {Z}_2$-trivial bands.

While Refs.^21,22,26–28^ advanced conceptual understanding of QSH effect, they relied on idealized models of decoupled Chern insulators, carrying opposite Chern numbers. Due to the underlying *U*(1) spin-rotation symmetry, these models admit $\mathbb {Z}$-classification of $\mathfrak {C}_{R,GS}=\mathfrak {C}_{GS,\uparrow }=-\mathfrak {C}_{GS,\downarrow }$, and the spin-Hall conductivity $\sigma ^z_{xy} = 2 \mathfrak {C}_{R,GS}$. The SCS for such systems would be governed by $SU(2)^{|\mathfrak {C}_{R,GS}|}$-multiplets [see supplementary material for example]. Since $\mathfrak {C}_{R,GS}$ for decoupled models is easily calculated, the analysis of SCS only serves academic interest.

For realistic band structures of spin-orbit-coupled materials, various crystalline-symmetry allowed hybridization terms destroy *U*(1) spin-conservation law. In the absence of *U*(1) spin-conservation, the spin-Chern can be calculated in the spin-subspace following the procedure put forth by Prodan^14^. However, this procedure can be computationally expensive and require detailed knowledge of the spin-operators, making it undesirable for large-scale application, particularly in an automated or high-throughput workflow. The insertion of a magnetic flux tube to probe SCS would allow unambiguous diagnosis of $|\mathfrak {C}_{R,GS}|$ or $\mathbb {N}$-classification of QSH insulators in a gauge-invariant and basis agnostic manner.

The outstanding need for a method with this capability is evident given the growing interest in two-dimensional insulators supporting even-integer $|\mathfrak {C}_{R,GS}|$^29,30^, as well as higher-order insulators with odd-integer $|\mathfrak {C}_{R,GS}|$ invisible to both the $\mathbb {Z}_{2}$ strong topological insulator index and Wilson loop computations^14,31,32^. Moreover, $\mathbb {Z}$-classification can be accomplished by measuring spin expectation values, during the process of spin-pumping.

Recently, we have addressed the stability of SCS for topologically non-trivial planes of 3D Dirac semimetals (4-band model)^32^, and 3-fold symmetric planes (8-band model)^33^ of octupolar topological insulators^34^. These models support SCS respectively controlled by *SU*(2) and $SU(2)^2$ multiplets. Moreover, the quantized pumping of spin occurs even in the absence spin-rotation symmetry and gapless, helical edge-states.

Encouraged by these results, in this work, we perform *proof of principle* demonstration of SCS for realistic, ab initio band structures. For concreteness, we focus on a single (111)-bilayer of elemental bismuth (Bi), also known as $\beta$-bismuthene, as a suitable material platform. The analysis of SCS will be guided by the calculation of gauge-invariant magnitudes of relative Chern numbers of constituent bands ($|\mathfrak {C}_{R,n}|$ for *n*-th band)^35^. Thus, the importance of $\mathbb {Z}_2$-trivial bands, possessing even integer $\mathfrak {C}_{R,j}$ will be critically addressed. We also present a brief contrasting study of $\beta$-antimonene, which does not exhibit SCS.

## Band topology of bismuthene

Symmetry-based topological classification of 3D Bi has dramatically evolved over past fifteen years. The band structure of Bi was initially classified as topologically trivial, with strong $\mathbb {Z}_2$ TI index $\nu _{0,GS}=0$^8,36^. Now it is identified as a higher-order, topological crystalline insulator with strong $\mathbb {Z}_4$ index $\kappa _{1,GS}= 2$^37–39^. In contrast to this, the ground state of $\beta$-bismuthene is known to be a $\mathbb {Z}_2$ QSH insulator^6,40–45^, supporting helical edge states^6,40,41^.

The crystal structure of $\beta$-bismuthene is described by buckled honeycomb layers with space group *P*6/*mcc*. The material supports $\mathcal {T}$ and space-inversion ($\mathcal {P}$) symmetries, leading to the two-fold Kramers degeneracy of all energy bands throughout the hexagonal BZ of Fig. 1a. Using the crystal structure and lattice constants from Ref.^46^, the ab initio band structure has been calculated with Quantum Espresso^47–49^. The band structure along high-symmetry path $\Gamma -M-K-\Gamma$ is shown in Fig. 1b. As these bands are well separated from other bands, an accurate 12-band, Wannier tight-binding (TB) model has been constructed from $p_{x,y,z}$ orbitals from each layer. We have included spin-orbit coupling for all calculations and utilized a 40 $\times$ 40 $\times$ 1 Monkhorst-Pack grid of $\varvec{k}$-points and a plane wave cutoff of 100 Ry. The model construction and topological analysis are performed with Wannier90 and Z2pack^17,19,50^.

When the Fermi level is tuned inside direct gaps between bands (i) 3 and 4, (ii) 4 and 5, and (iii) 5 and 6, we find three insulators at filling fractions 1/2, 2/3, and 5/6. From the parity eigenvalues shown in Fig. 1b, we find that only bands 2 and 6 possess non-trivial $\mathbb {Z}_2$-index $\nu _{0,n}=1$, and all three insulators admit non-trivial $\mathbb {Z}_2$-index $\nu _{0,GS}=1$. The results of edge-states calculations, using iterative Greens function method^51^ and Wannier Tools^52^ are displayed in Fig. 1c,d. All three insulators support gapless edge modes, which can cross the Fermi level 2 or 6 times^40^. Whether $|\mathfrak {C}_{R,GS}|=1$ or 3 cannot be determined from edge-spectrum.

It is instructive to compute spin Hall conductivity, following the current state-of-the-art of computational materials science, Ref.^53–55^ and the results are shown in Fig. 2. Due to the non-conservation of spin, this method cannot capture quantization of spin Hall effect. But it provides rough guidance for understanding qualitative properties of three insulators. Notice that $\sigma ^z_{xy}$ displays plateau-like features, when *E* is tuned in direct band gaps, and changes sign when $\mathbb {Z}_2$-trivial bands 4 and 5 become occupied. Are bands 4 and 5 topologically trivial?

**Table 1:** Natural number classification of relative Chern numbers of constituent Kramers-degenerate bands of $\beta$-bismuthene.

index *n*	Threefold eigen-values $C_{3,n}$	$\mathbb {Z}_2$-index $\nu _{0,n}$	Relative Chern number $|\mathfrak {C}_{R,n}|$
1	$e^{\pm i \frac{\pi }{3}}$	0	0
2	$e^{\pm i \frac{\pi }{3}}$	1	1
3	$e^{\pm i \frac{\pi }{3}}$	0	0
4	$e^{\pm i \pi }$	0	2
5	$e^{\pm i \frac{\pi }{3}}$	0	2
6	$e^{\pm i \pi }$	1	1

The threefold rotation eigenvalues and $\mathbb {Z}_2$-indices are listed for convenience. The symmetry data of rotation and parity eigenvalues are insufficient to distinguish between bands possessing $|\mathfrak {C}_{R,n}|=0$ and 2.

**Figure 3:**
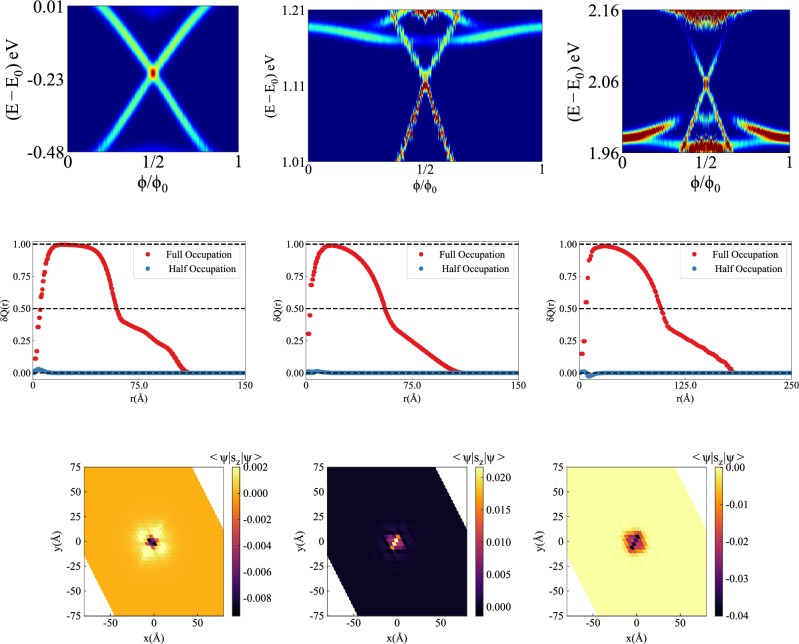
Spin-charge separation in $\beta$-bismuthene. (**a–c**) Local density of states on flux tube, as a function of $\phi /\phi _0$, with energies scanned in the vicinity of band gaps. For time-reversal-invariant flux $\phi =\phi _0/2$, twofold degenerate bound states occur precisely at 1/2, 2/3, and 5/6 filling fractions. For any generic value of flux, time-reversal-symmetry is broken and bound states become non-degenerate. As $\phi$ is tuned from 0 to $\phi _0$, one Kramers-pair is pumped, revealing $|\mathfrak {C}_{R,GS}|=1$ for all three insulators. Induced electric charge $\delta Q(r)$ (in units of $-e$) on flux tube, as a function of radial distance *r* from the flux tube, for (**d**) 1/2-filled, (**e**) 2/3-filled, (**f**) 5/6-filled insulators, with $\phi /\phi _0 =1/2$. When the bound states are half-filled (fully occupied), the maximum value of induced charge saturates to quantized value 0 ($-e$). The results remain unchanged for $\phi /\phi _0 \ne 1/2$. The spin expectation values of unoccupied bound modes for $\phi =(\frac{1}{2}-\epsilon )\phi _{0}$, and $\epsilon =10^{-3}$ for (**g**) 1/2-filled, (**h**) 2/3-filled, (**i**) 5/6-filled insulators. The profile of spin polarization for 2/3-filled insulator is opposite to those for 1/2- and 5/6- filled insulators.

This question can be conclusively answered by identifying the magnitude of relative Chern number of Kramers-degenerate bands ($|\mathfrak {C}_{R,n}|$). This is accomplished using two separate methods. First we consider computation of in-plane Wilson loops of *SU*(2) Berry connection^35^. The results are displayed in Table 1 and the details of the calculations are presented in the Supplementary Material. We see that bands 4 and 5 carry even integer invariants. While they do not change odd integer classification of $\mathfrak {C}_{R, GS}$, they can change the magnitude and the sign of $\mathfrak {C}_{R, GS}$. Table 1 suggests the following possibilities: (i) $|\mathfrak {C}_{R, GS}| = 1$, (ii) $|\mathfrak {C}_{R, GS}|=1,3$, (iii) $|\mathfrak {C}_{R, GS}|=1,3,5$, respectively for 1/2, 2/3, and 5/6 filled insulators. To verify these results and probe the additive nature of the relative Chern number defined for each Kramers degenerate band, we compute the spin-resolved Wilson loop following the method set forth by Prodan^14^. This method is advantageous as it can be directly applied to sets of occupied bands. A detailed description of the method is presented in the Supplementary Material as well as the results which demonstrate $|\mathfrak {C}_{R, GS}| = 1$ for 1/2, 2/3, and 5/6 filled insulators. We emphasize that although the spin-resolved Wilson loop is a powerful method, it is not well suited for complex materials of many bands or automated workflows given the need to identify a preferred spin-direction. In the next section, we resolve $|\mathfrak {C}_{R, GS}|$ in a basis agnostic manner via thought experiments with flux tubes.

## Spin charge separation

To study real-space topological response, we insert a flux tube at the center of 2D system. The hopping matrix element $H_{ij}$, connecting lattice sites $\varvec{r}_i$ and $\varvec{r}_j$ is modified to $H_{ij} \; e^{i \phi _{ij}}$. Working in Coulomb gauge, we define the Peierls phase factor

1 $$\begin{aligned} \phi _{ij}= \frac{\phi }{\phi _0} \int _{\varvec{r}_i}^{\varvec{r}_j} \frac{\hat{z} \times \varvec{r}}{\varvec{r}^2} \cdot d\varvec{l}. \end{aligned}$$

We first perform exact diagonalization of the gauged Hamiltonian for $24 \times 24$ unit cells, under periodic boundary conditions (PBC), yielding $N=6912$ eigenstates. When the total number of electrons $N_e=\frac{N}{2}, \frac{2N}{3}, \frac{5N}{6}$, and $\phi =\phi _{0}/2$, we find two-fold-degenerate mid-gap states bound to the flux tube, leading to two-fold degeneracy of ground states (see Fig. 3a–c). When $\phi \ne \phi _0/2$, flux tube breaks $\mathcal {T}$-symmetry and the degeneracy of bound states, and the ground states is lifted. By holding $N_e$ fixed at commensurate values, and varying $\phi$ from 0 to $\phi _0$, we observe pumping of one Kramers pair^21^.

Next we compute the induced electric charge on flux-tube for $\phi =\phi _{0}/2$. This calculation is done in two steps^56^. For a given number of electrons, by summing over *all occupied states*, we evaluate the area charge densities $\sigma _1(\varvec{r}_i, N_e)$, and $\sigma _0(\varvec{r}_i, N_e)$, respectively in the presence and absence of flux tube. The induced charge density is defined as $\delta \sigma (\varvec{r}_i, N_e) = \sigma _1(\varvec{r}_i, N_e)-\sigma _0(\varvec{r}_i, N_e)$, and the total induced charge within a circle of radius *r*, centered at the flux tube is determined from $\delta Q(r,N_e)=\sum _{|\varvec{r}_i|<r}\delta \sigma (\varvec{r}_i, N_e)$. In order to achieve sufficient numerical accuracy, induced charge calculations are performed for a system size of $60 \times 60$ unit cells, yielding $N=43,200$ eigenstates. The results are displayed in Fig. 3d–f. When $N_e=\frac{N}{2}, \frac{2N}{3}, \frac{5N}{6}$, one of the degenerate mid-gap modes is occupied (half-occupation of mid-gap states), and we find $\delta Q(r,N_e) =0$, which remains unchanged for generic values of $\phi$. For $N_e= \frac{N}{2} \pm 1, \frac{2N}{3} \pm 1, \frac{5N}{6} \pm 1$, the bound modes become completely occupied ($+$) and empty (−), and the maximum values of $\delta Q (r, N_e)$ saturate to quantized results ${\mp }e$, respectively.

Therefore, we can conclude that each non-trivial insulator supports ${|}{\mathfrak {C}}_{{R},{GS}}{|}=1$, which can only be consistent with the following assignments of signed relative Chern numbers

2 $$\begin{aligned}{} & {} (\mathfrak {C}_{R, 1},\mathfrak {C}_{{R}, {2}},\mathfrak {C}_{R, 3},\mathfrak {C}_{R, 4},\mathfrak {C}_{R, 5},\mathfrak {C}_{R, 6}) \nonumber \\{} & {} = \pm (0, 1,0,-2,+2,-1), \end{aligned}$$

defined with respect to a global spin quantization axis for all bands. When bands 4 and 5 are occupied, $\mathfrak {C}_{{R},{GS}}$ will change sign. This change in sign of $\mathfrak {C}_{{R},{GS}}$ at each filling fraction can be further substantiated by evaluating expectation values of the bound states for the spin operator, $\hat{s}_{z}$, utilized in computation of $C_{{R},{GS}}$ via spin-resolved Wilson loop. While further details of the spin-resolved Wilson loop itself are available in Sect. II and the Supplementary Material, the expectation value of $\hat{s}_{z}$ for the mid-gap bound modes provides information regarding the relative sign of $C_{{R},{GS}}$ at distinct filling fractions due to the vortex acting as a spin-pump.

As an example, consider an insulator supporting $\mathfrak {C}_{{R},{GS}}=+1$ at a given filling fraction. Suppose further that the spin-expectation value, along the preferred spin-direction, of the occupied bound state at $\phi =(\frac{1}{2} - \epsilon )\phi _{0}$ is positive. If at a separate filling fraction this insulator admits $\mathfrak {C}_{{R},{GS}}=-1$ then the behavior of the spin-pump must be reversed. In correspondence, the expectation value of the preferred spin-direction for the bound mode at $\phi =(\frac{1}{2} - \epsilon )\phi _{0}$ must reverse its sign. We further elaborate on this relationship for analytically known models in the supplementary material.

We have computed expectation values for $\phi =(\frac{1}{2} - \epsilon )\phi _{0}$ and $\epsilon \rightarrow 0^{+}$, such that the bound states are infinitesimally split in energy in $\beta$-bismuthene. As occupied and unoccupied modes support opposite signs for $\left\langle \psi _{n}\right| s_{z} \left| \psi _{n}\right\rangle$, we are only showing the results for unoccupied modes in Fig. 3g–i. We note a clear inversion in the behavior of the expectation value density at each subsequent filling fraction, demonstrating a reversal in the behavior of the spin-pump. This is in accordance with $\mathfrak {C}_{{R},{GS}}$ alternating sign for $1/2-,2/3-, \text {and} 5/6$-filled insulators.

## Conclusions

In summary, we have shown that bilayer bismuth is a suitable platform for studying spin charge separation as a universal topological response of quantum spin Hall insulators. The combined analysis of non-Abelian Berry connection in momentum space and real-space topological response revealed important role of topologically non-trivial bands that carry even integer $\mathfrak {C}_{{R},{GS}}$. We have also checked the robustness of our findings for spin-charge separation by incorporating remote valence bands. The density functional theory predicts a total number of fifteen two-fold degenerate valence bands. Our results for spin-charge separation for three distinct quantum spin-Hall insulators were reproduced by probing a 36-band tight binding model. In the supplementray material we present a contrasting study of bulk topology of $\beta$-antimonene, which supports a $\mathbb {Z}_2$-trivial ground state. With the first principles based calculations of spin Hall conductivity and the insertion of magnetic flux, we show that $\beta$-antimonene is not a quantum spin Hall insulator ( i.e., $\mathfrak {C}_{{R},{GS}}=0$).

In this work, we have only gauged a conserved quantity (electric charge) to identify the presence or absence of spin-pumping. This can be reliably used for many candidate materials for quantum spin Hall effect. We emphasize the necessity of this method as it goes beyond symmetry indicators and calculations of Wannier center charge spectra, yielding the precise bulk invariant even in the absence of *U*(1) spin-conservation symmetry and/or inversion symmetry. Guided by the results of this work, one can further pursue insertion of spin-gauge flux (gauging of non-conserved quantity) to demonstrate pumping of electric charge ($\delta Q= 2 e \mathfrak {C}_{{R},{GS}}$), which directly tracks signed relative/spin Chern number^21,57^. Due to technical subtleties and numerical cost of such calculations, such thought experiments on real materials would be reported in a future work.

## Supplementary Information

Supplementary Information.

## Data Availability

The datasets used and/or analysed during the current study available from the corresponding author on reasonable request.
