# Supplementary material for: Spin-charge separation and quantum spin Hall effect of β-bismuthene
Source: Sci Rep. 2023 Jul 14;13:11393. doi: 10.1038/s41598-023-38491-1 (PMC10349063; doi:10.1038/s41598-023-38491-1)
Supplement: Supplementary file 1 — Supplementary Information. [file 41598_2023_38491_MOESM1_ESM.pdf]

Supplementary Material: Spin-charge separation and quantum spin Hall effect of β-bismuthene

Alexander C. Tyner[1] and Pallab Goswami[1,2]
[1] Graduate Program in Applied Physics, Northwestern University, Evanston, Illinois, 60208, USA and
[2] Department of Physics and Astronomy, Northwestern University, Evanston, Illinois, 60208, USA
(Dated: June 17, 2023)

I. SPIN-CHARGE SEPARATION OF MINIMAL MODELS

Let us consider the following form of Bernevig-Hughes-Zhang (BHZ) model [1] of sp hybridized topological insulators on a square lattice

$$\frac{H_0(\mathbf{k})}{t} = \sum_{j=1}^{3} d_j(\boldsymbol{k})\Gamma_j = \Delta_0 \sin k_x \Gamma_1 + \Delta_0 \sin k_y \Gamma_2 + [M + \Delta_1(\cos k_x + \cos k_y) + \Delta_2 \cos k_x \cos k_y]\Gamma_3, \tag{S1}$$

where $\Gamma_1 = \tau_x \otimes \sigma_z$, $\Gamma_2 = \tau_y \otimes \sigma_0$, and $\Gamma_3 = \tau_z \otimes \sigma_0$ are mutually anti-commuting matrices. The 2×2 identity matrix σ_0 (τ_0) and Pauli matrices $\sigma_{j=x,y,z}$ ($\tau_{j=x,y,z}$) operate on the spin (orbital/parity) index. The hopping parameter t has units of energy, and M, Δ_0, Δ_1, Δ_2 are dimensionless tuning parameters, and the lattice constant has been set to unity. The Hamiltonian anti-commutes with $\Gamma_4 = \tau_x \otimes \sigma_x$, and $\Gamma_5 = \tau_x \otimes \sigma_y$, and commutes with $\Gamma_{45} = [\Gamma_4, \Gamma_5]/(2i) = \tau_0 \otimes \sigma_z$. Thus, $\Psi^\dagger(\boldsymbol{r}_i)\mathbb{1}\Psi(\boldsymbol{r}_i)$ and $\Psi_i^\dagger \Gamma_{45}\Psi$ are the generators of $U_+(1)$ and $U_-(1)$ symmetries, which respectively describe the total number and the spin (total S_z) conservation laws.

For two-dimensional systems, the space-inversion operation $\boldsymbol{k} \to -\boldsymbol{k}$ is equivalent to the two-fold rotation iC_{2z}. Since $\Gamma_3 H_0(-\boldsymbol{k})\Gamma_3 = H_0(\boldsymbol{k})$, Γ_3 represents the space-inversion or parity operator $\mathcal{P}$. At the time-reversal-invariant momentum points $\Gamma : \boldsymbol{Q} = (0,0)$, $M : \boldsymbol{Q} = (\pi,\pi)$, and $X : \boldsymbol{Q} = \{(\pi,0),(0,\pi)\}$ points $H_0(\boldsymbol{Q}) = t d_3(\boldsymbol{Q})\Gamma_3$, and $[H_0(\boldsymbol{Q}), \mathcal{P}] = 0$. The parity eigenvalues of valence bands are given by $-\mathrm{sgn}(d_3(\boldsymbol{Q}))$. A representative phase diagram is shown in Fig. S1, and the pattern of parity eigenvalues and the bulk invariant

$$\mathfrak{C}_{R,GS} = \frac{1}{4\pi}\int_{BZ} d^2k\, \hat{\boldsymbol{d}} \cdot \left(\frac{\partial \hat{\boldsymbol{d}}}{\partial k_x} \times \frac{\partial \hat{\boldsymbol{d}}}{\partial k_y}\right) \tag{S2}$$

are listed in Table S1. After tracing over two spin projections of occupied valence bands we arrive at the spin Chern number $\mathfrak{C}_s = \mathfrak{C}_{R,GS}$.

As the phases 3, 4, 6, and 7 (2 and 5) support $|\mathfrak{C}_{R,GS}| = 1$ ($|\mathfrak{C}_{R,GS}| = 2$), magnetic π-flux tube would bind 2 (4) mid-gap states. In the case of $|\mathfrak{C}_{R,GS}| = 1$, SCS is then controlled by the $SU(2)$ doublet. For $|\mathfrak{C}_{R,GS}| = 2$, we find that the sp hybridization allows for a crystalline doublet that protects the four-fold degeneracy of the mid-gap states and leads to the SCS being described an $SU(4)$ multiplet, (see Fig. S2). We emphasize that for a generic model, lacking the protection of the crystalline doublets, and preserving only time-reversal symmetry, mid-gap modes form $SU(2)^{|\mathfrak{C}_{R,GS}|}$ doublets. An example of this is given in Fig. (S4).

After Fourier transformation, we obtain tight-binding model $H_{0,ij}$ in real-space. In the presence of magnetic flux tube, placed at origin, the matrix elements $H_{0,ij}$ between different lattice sites can be replaced by $H_{0,ij}e^{i\phi_{ij}}$, with $\phi_{ij} = \frac{\phi}{\phi_0}\int_{\boldsymbol{r}_i}^{\boldsymbol{r}_j} \frac{\hat{z}\times\boldsymbol{r}}{\boldsymbol{r}^2}\cdot d\boldsymbol{l}$. The SCS and spin-pumping for Phases 3, 4, 6, and 7 are controlled by $SU(2)$ multiplets. In Fig. S3, we show the results for Phase 3 and Phase 4.

The results of SCS for Phases 2 and 5 are displayed in Fig. S4, clearly showing that 2 Kramers-pair are being pumped. Due to the enhanced degeneracy of bound states, the maximum induced electric charge can now oscillate between $0, \pm e$, and $\pm 2e$.

Spin-pumping: As the flux, ϕ, is tuned from 0 to ϕ_0 in an insulator with non-zero spin Chern number, spin is pumped across the bulk gap. In the case of $U(1)$ spin-conservation law along spin axis $\hat{s}_z$, spin is pumped

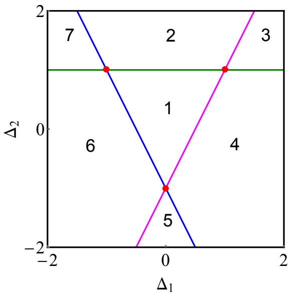

FIG. S1. Phase diagram of four-band model for $M = 1$. Parity eigenvalues and bulk winding numbers are listed in Table S1. Along blue, magenta, and green lines, the bulk band gap can close at Γ, M, and X points, respectively. Red dots denote multi-critical points.

in quantized units as measured through computation of the spin expectation value $\langle\psi|\, \hat{s}_z \,|\psi\rangle$ for the occupied subspace. This can be seen by noting that $\langle\psi|\, \hat{s}_z \,|\psi\rangle = 0$ for the occupied subspace when $\phi = 0$. As we tune ϕ, suppose $\mathfrak{C}_{R,GS} > 0$ and an up-spin state is pumped from the occupied to unoccupied subspace, leading to the occupied subspace acquiring $\langle\psi|\, \hat{s}_z \,|\psi\rangle = -2$ when $\phi = \phi_0$. If in the same system we tune parameters such that $\mathfrak{C}_{R,GS} < 0$, the behavior of the spin-pump must be reversed. Correspondingly, the state pumped from the occupied to unoccupied subspace must be a down-spin state. If $U(1)$ spin-conservation is broken, the quantity of spin pumped remains finite but is no longer quantized. In this manner, it is clear that we can probe the sign of the spin-Chern number through computation of the spin-expectation value for the vortex bound modes at $\phi = \phi_0(\frac{1}{2} - \epsilon)$. In Fig. (S3), we demonstrate this by computing the spin expectation value for the vortex bound modes as well as the ground state spin-expectation value for phases 3 and 4.

In an experiment, it is possible to alter the strength of spin-orbit coupling or change the preferred spin direction. In these cases, assignment of the signed invariant will break down. However, we are considering a numerical model for which the preferred spin direction and spin gap is fixed, protecting assignment of the integer valued invariant.

Absence of $U(1)$ spin-conservation symmetry: There are many ways to break $U_-(1)$ spin rotation symmetry. For example, we can modify H_j as

$$H_0(\mathbf{k}) \to H(\mathbf{k}) = H_0(\mathbf{k}) + d_4(\boldsymbol{k})\Gamma_4 + d_5(\boldsymbol{k})\Gamma_5, \tag{S3}$$

such that the 2-fold Kramers-degeneracy is preserved. The momentum dependent function $d_4(\mathbf{k}) = t_{d,1}(\cos 2k_x - \cos 2k_y)$ and $d_5(\boldsymbol{k}) = t_{d,2} \sin k_x \sin k_y$ maintain 4-fold rotation symmetry. Following Ref. 2, it can be shown that $\mathfrak{C}_{R,GS}$ and SCS for all phases remain unchanged. But d-wave perturbations destroy *gapless edge-states.*

We can further utilize eq. (S3) to demonstrate the existence of $SU(2)^{|\mathfrak{C}_{R,GS}|}$ doublets for $|\mathfrak{C}_{R,GS}| = 2$ in the absence of enhanced crystalline symmetry. This is accomplished by fixing $\Delta_0 = 0$, $\Delta_1 = 1$,$\Delta_2 = 0$ and $t_{d,j=1,2} = t$. The resulting model of an *sd* hybridized topological insulator on a square lattice can then be shown to support $|\mathfrak{C}_{R,GS}| = 2$ via eq. (S2). Insertion of the magnetic flux tube leads to the results shown in Fig. (S4) with SCS being governed by $SU(2)^2$ doublets.

An example of decoupled models with higher number of bands can be found in Ref. 3. Using a model of three Kramers degenerate bands on the Kagome lattice, with $U(1)$ spin-rotation symmetry, Wang *et. al.* found $(\mathfrak{C}_{R,1}, \mathfrak{C}_{R,2}, \mathfrak{C}_{R,3}) = (-1, +2, -1)$. Consequently, $C_{R,GS} = -1, +1$ for 1/3- and 2/3- filled insulators, and both states exhibited SCS governed by $SU(2)$ multiplets. This situation is similar to our observations in β-bismuthene.

II. MAGNITUDE OF RELATIVE CHERN NUMBERS

A. Computation via in-plane $SU(2)$ Wilson loop

Recently, the in-plane Wilson loop has been utilized to quantify magnitude of $SU(2)$ Berry flux of constituent Kramers-degenerate bands of two-dimensional first and higher-order topological insulators. [2, 4]

Phase	Parity eigenvalues $(\delta_\Gamma, \delta_M, \delta_X)$	$\mathfrak{C}_{R,GS}$
1	$(-1,-1,-1)$	0
2	$(-1,-1,+1)$	$2\ \mathrm{sgn}(t)$
3	$(-1,+1,+1)$	$\mathrm{sgn}(t)$
4	$(-1,+1,-1)$	$-\mathrm{sgn}(t)$
5	$(+1,+1,-1)$	$-2\ \mathrm{sgn}(t)$
6	$(+1,-1,-1)$	$-\mathrm{sgn}(t)$
7	$(+1,-1,+1)$	$\mathrm{sgn}(t)$

TABLE S1. Patterns of parity eigenvalues and bulk winding numbers for various phases of Fig. S1.

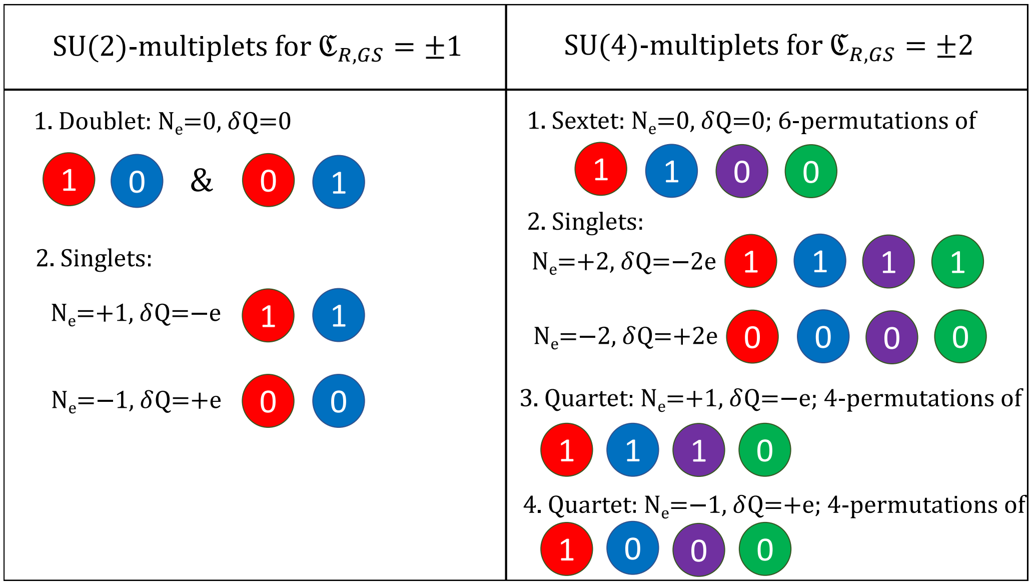

FIG. S2. Schematic of spin-charge separation and induced quantum numbers for magnetic π flux tube. The occupation number of mid-gap states is denoted by 0 and 1. For half-filled systems, the number of added electrons $N_e = 0$, and the induced electric charge $\delta Q = 0$. By adding one electron or hole one can access $\delta Q = \mp e$ on flux tube for $\mathfrak{C}_{R,GS} = \pm 1$. Enhanced symmetry can protect an $SU(4)$ multiplet for $\mathfrak{C}_{R,GS} = \pm 2$, increasing the degeneracy of the possible configurations. In generic systems, additional charge quantum numbers can be realized, but their enhanced degeneracy is lost.

The in-plane Wilson loop of n-th band measures $SU(2)$ Berry phase accrued upon parallel transport along a non-intersecting closed contour C. It is defined by

$$W_n = P \exp\left[i \oint A_{j,n}(\mathbf{k}) dk_j\right] = \exp\left(i\theta_n \hat{\mathbf{\Omega}}_n \cdot \sigma\right), \tag{S4}$$

where $A^{ss'}_{j,n}(\mathbf{k}) = -i\langle\psi_{n,s}(\boldsymbol{k})|\partial_j \psi_{n,s'}(\boldsymbol{k})\rangle$ describes components of $SU(2)$ Berry connection, $\partial_j = \frac{\partial}{\partial k_j}$, $\psi_{n,s=\pm1}(\mathbf{k})$ are degenerate eigenfunctions of n-th band, and $\mathcal{P}$ indicates path-ordering. While the angle θ_n measures gauge-invariant magnitude of non-Abelian flux enclosed by C, the three-component unit vector $\hat{\mathbf{\Omega}}_n$ depends on gauge choice.

Following the convention of defining gauge-invariant eigenvalues of Wilson lines or Wannier center charges, we analyze eigenvalues of $\mathrm{Im}(\mathrm{Ln}(W_n)) \equiv \pm|\theta_n| \mod \pi$. In-plane loops are calculated with Wannier90 [5] and Z2Pack software packages, by following C_3-symmetry preserving contour, shown in Fig. S5a. The area enclosed by the contour is systematically increased from zero to the area of first Brillouin zone. The number

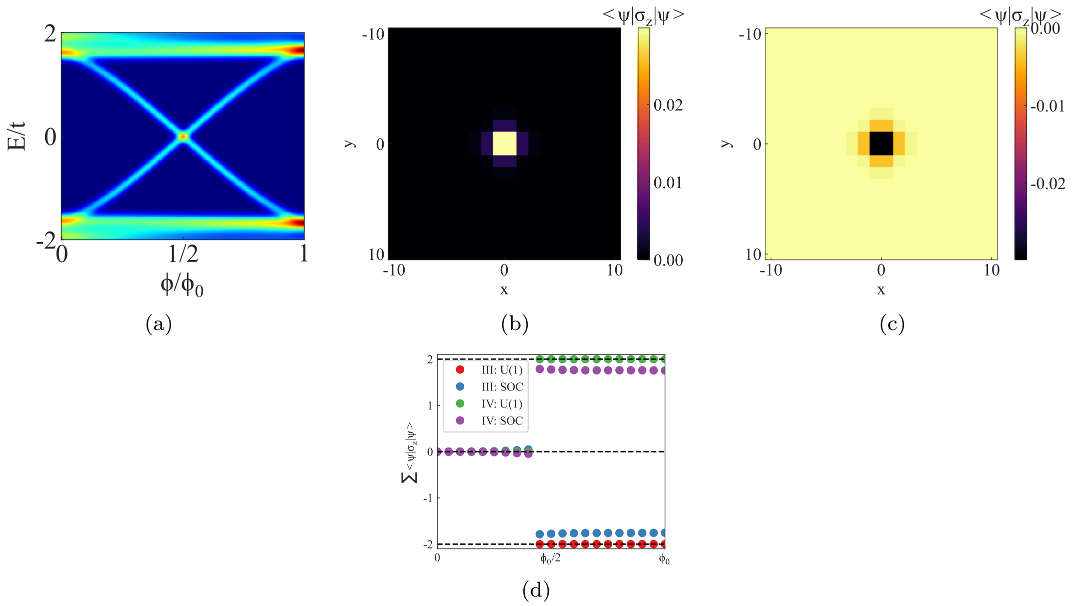

FIG. S3. Spin-charge separation for Phase 3 and Phase 4, with $t > 0$. (a) Local density of states on flux tube for both phases shows pumping of one Kramers pair. Spin expectation value $\langle\psi_n|\,\sigma_z\,|\psi_n\rangle\,(x, y)$ of unoccupied bound mode, when $\phi = (\frac{1}{2} - \epsilon)\phi_0$, and $\epsilon = 10^{-2}$, for (b) Phase 3, (c) Phase 4. The spin density on flux tube tracks $\mathrm{sgn}(\mathfrak{C}_{R,GS})$. (d) Sum of expectation value of the spin-operator $\hat{\sigma}_z$ for all occupied states as a function of flux strength for phase 3 and 4, in the presence of $U(1)$ spin conservation law and in the presence of spin-orbit coupling (SOC), when $t_{d,j=1,2} = 1/2$.

of winding of θ_n corresponds to the absolute value of relative Chern number $|\mathfrak{C}_{R,n}|$. The results for bands 1-6 are shown in Fig. S5b-S5g.

B. Computation via spin-resolved Wilson loop

We could have also computed the relative Chern numbers following the procedure outlined by Prodan[6]. This procedure requires defining the projected spin operator (PSO), $P(\mathbf{k})\hat{s}P(\mathbf{k})$, where $P(\mathbf{k})$ is the projector onto a set of bands and $\hat{s}$ is a chosen traceless operator. In general, $\hat{s}$ is the preferred spin-direction, however, Prodan notes that it can in principle be any traceless operator which results in a gapped spectra for the PSO, such that a ground-state Chern number can be computed for the negative (occupied) subspace of the PSO. In the absence of spin-orbit coupling the eigenvalues of the PSO are fixed as ± 1. Since β-bismuthene admits spin-orbit coupling, the eigenvalues are no longer pinned at ± 1.

It is computationally challenging to acquire complete knowledge and control of the basis for the maximally localized Wannier tight-binding model. Nevertheless, we scan the possible traceless operators to identify the operator which supports a spin-gap, which we denote $\hat{s}_z$. Utilizing this operator to form the PSO, we find the resulting values of the relative Chern number, defined for each band, is in direct correspondence with the gauge-invariant magnitude computed via $SU(2)$ Wilson loop. The explicit form of the operator is $\hat{s}_z = \mathbb{1}_{6\times 6} \otimes \sigma_3$ for our model in a basis for which the Bloch Hamiltonian is diagonal at the Γ point, taking the form $H = \mathrm{diag}\,(E_1, E_1, E_2, E_2, ...E_6, E_6)$, where E_n is the eigenvalue of band n.

The primary benefit of this method, is that upon identification of $\hat{s}$, the relative Chern number can be computed for a set of occupied bands to determine the additive nature of the invariant computed for individual Kramers pairs. To this end, in Fig. S5h-S5l we compute the spin resolved Wilson loop considering bands $1 - N$ to be occupied, resulting in $C_{R,N/6}$ for $N \in \{1, 2, 3, 4, 5\}$. The results demonstrate that the magnitude of relative Chern number at each value of N is in agreement with the value of $|\mathfrak{C}_{R,GS}|$ as determined from a combined analysis of flux insertion and $SU(2)$ Wilson loop. Furthermore, we note a change in sign for the invariant when $N : 3 \to 4$ and $N : 4 \to 5$.

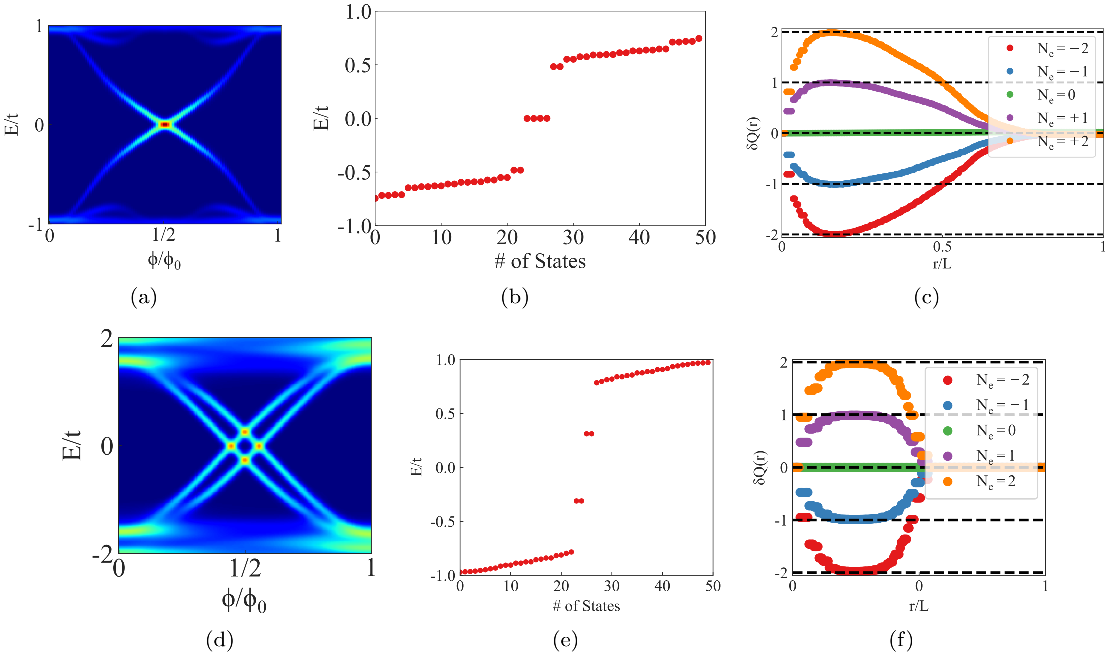

FIG. S4. Spin-charge separation for Phase 2 and Phase 5, possessing $|\mathfrak{C}_{R,GS}| = 2$. All calculations are performed for a system size of 24×24 lattice sites, under periodic boundary conditions. (a) Local density of states on flux tube as a function of ϕ/ϕ_0. Both branches of spectra, which traverse the bulk gap are two-fold degenerate, implying pumping of two Kramers pairs. This degeneracy is protected by the fact that the sp hybridized model supports crystalline doublets. In the absence of this enhanced symmetry, the degeneracy of the branches is broken, as seen in (d) for which we have fixed $\Delta_{0,2} = 0$, $\Delta_1 = 1$ and $t_{d,j=1,2} = t$. At $\phi = \phi_0/2$, we find four mid-gap bound states. The $SU(4)$ multiplet shown in (b) realizes 6-fold degeneracy of the half-filled ground state. However, in a generic model, we find $SU(2)^{|\mathfrak{C}_{R,GS}|}$ doublets, as seen in (e). Induced electric charge (in units of $-e$) can be calculated on π-flux tube within a radius r, and N_e denotes the number of doped electrons for both systems, yielding the identical results seen in (c) and (f).

III. ANALYSIS OF β-ANTIMONENE

In contrast to β-bismuthene, the occupied subspace of single layer of (111) antimony (β-antimonene) supports trivial $\mathbb{Z}_2$-classification with $\nu_{0,GS} = 0$. Whether the ground state supports quantum spin Hall effect can be directly addressed by combined analysis of momentum space topology and real-space response. The bulk band structure and $\mathbb{N}$-classification of constituent bands are shown in Fig. S6a and Fig. S6b, respectively. We have used the lattice parameters given by Mounet *et. al.* [7]. Since occupied bands $n = 1, 2, 3$ possess $|\mathfrak{C}_{R,n}| = 0, 1, 1$, $|\mathfrak{C}_{R,GS}| = 0, 2$ are two possible options for the net relative Chern number.

The first principles calculations demonstrate that all three components of spin Hall conductivity vanish for the half-filled insulating state, as shown in Fig. S6c. To unambiguously probe topological response, we have performed thought experiments with flux tube for a system size of 24×24 unit cells, under periodic boundary conditions. The spectrum, shown in Fig. S6d, does not show any mid-gap bound states for $N_e = N/2$ and no spin-pumping is observed, implying $\mathfrak{C}_{R,GS} = 0$. Therefore, $(\mathfrak{C}_{R,2}, \mathfrak{C}_{R,3}) = \pm(1, -1)$ are the possible assignments of signed relative Chern numbers. Due to the lack of any further direct band gaps, we do not pursue the analysis for other filling fractions.

---

[1] Bernevig, B., Hughes, T., and Zhang, S.-C., "Quantum spin Hall effect and topological phase transition in HgTe quantum wells," Science **314**, 1757–1761 (2006).

[2] Tyner, A., Sur, S., Puggioni, D., Rondinelli, J. M., and Goswami, P., "Topology of three-dimensional dirac

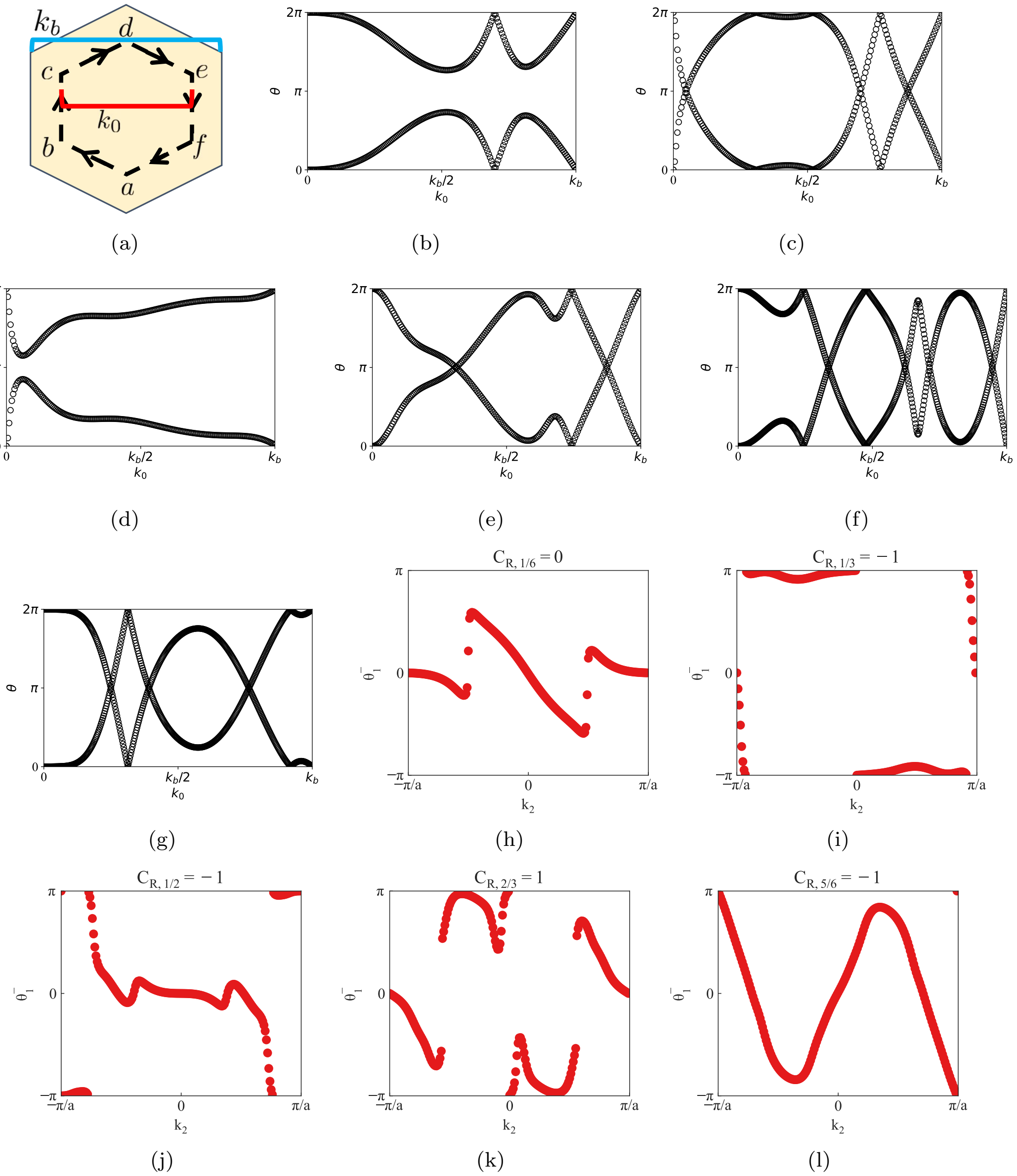

FIG. S5. (a) Schematic of path $(abcdef)$ for calculating in-plane Wilson loop. The size k_0 is increased from 0 to k_b. The results for bands 1-6 are shown in (b)-(g), respectively. Trivial bands 1 and 3 do not show winding of θ. For bands 2 and 6, possessing non-trivial $\mathbb{Z}_2$ index, θ winds once. For $\mathbb{Z}_2$-trivial bands 4 and 5, θ winds twice. Therefore, bands 1 through 6 support relative Chern numbers $|\mathfrak{C}_{R,n}| = 0, 1, 0, 2, 2, 1$, respectively.(h)-(l) Results of the spin-resolved Wilson loop, demonstrating the Wilson loop winding for Wannier centers of the negative bands of the projected-spin operator, θ_1^-, as a function of transverse momenta k_2 for filling fraction $N/6$ with $N \in \{1, 2, 3, 4, 5\}$, respectively. We note the ground-state relative Chern number, $\mathfrak{C}_{R,N/6}$, changes sign for for $N : 3 \to 4$ and $N : 4 \to 5$, in agreement with the analysis accomplished via flux insertion. The relative Chern number is defined precisely up to a global sign change.

semimetals and quantum spin hall systems without gapless edge modes," Phys. Rev. Res. **5**, L012019 (2023).

[3] Wang, Z. and Zhang, P., "Quantum spin Hall effect and spin-charge separation in a kagomé lattice," New Journal of Physics **12**, 043055 (2010).

[4] Tyner, A. *et al.*, "Quantized non-abelian, Berry's flux and higher-order topology of Na_3Bi," arXiv:2102.06207 (2021).

[5] Pizzi, G. *et al.*, "Wannier90 as a community code: new features and applications," J. Phys. Condens. Matter **32**,

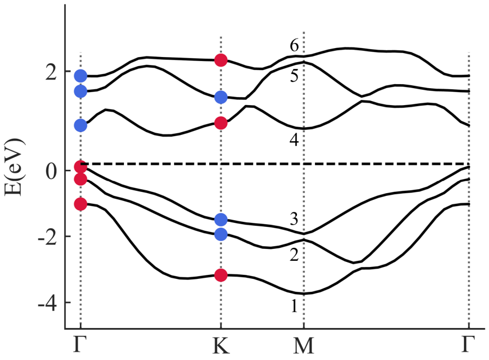

(a)

Band	$\nu_{0,n}$	$C_{3,n}$	$\lvert\mathfrak{C}_{R,n}\rvert$
1	0	$e^{\pm i\pi/3}$	0
2	1	$e^{\pm i\pi/3}$	1
3	1	$e^{\pm i\pi}$	1
4	1	$e^{\pm i\pi/3}$	1
5	0	$e^{\pm i\pi/3}$	2
6	1	$e^{\pm i\pi}$	1

(b)

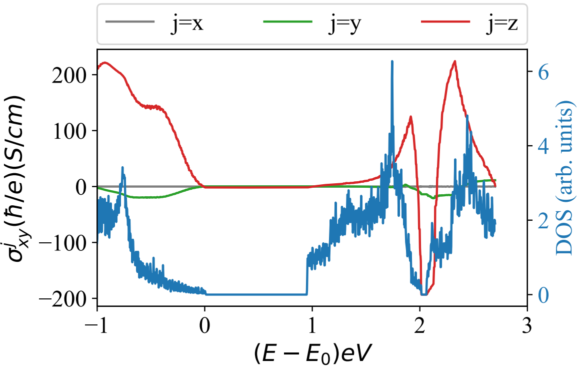

(c)

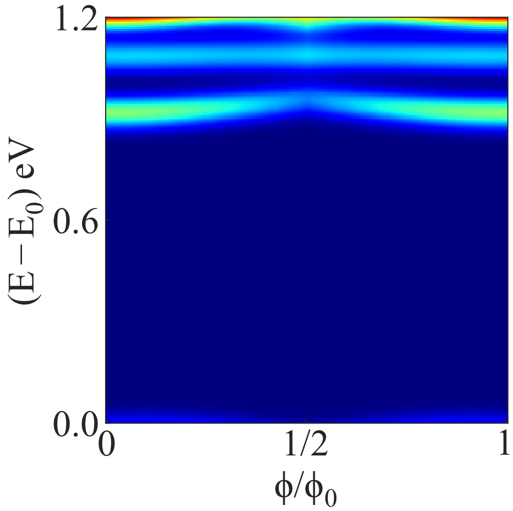

(d)

FIG. S6. (a) Band structure of β-antimonene, along high-symmetry path of hexagonal Brillouin zone. The bands are numbered according to their energies at Γ point, and parity eigenvalue $+1$ (-1) at time-reversal-invariant momentum points are denoted by red (blue) dots. (b) Summary of momentum-space topology of constituent bands, where $\nu_{0,n}$, $C_{3,n}$, and $\mathfrak{C}_{R,n}$ respectively denote the $\mathbb{Z}_2$ index, 3-fold rotation eigenvalue, and the relative Chern number of n-th Kramers-degenerate bands. (c) First principle calculations of spin Hall conductivity. When the Fermi level is tuned inside direct band gap, all three components of spin Hall conductivity vanish for the insulating state. (d) Local density of states on the magnetic flux tube in the vicinity bulk band gap does not show any spin-pumping, which shows that the net relative Chern number $\mathfrak{C}_{R,GS} = 0$.

165902 (2020).
[6] Prodan, E., "Robustness of the spin-chern number," Phys. Rev. B **80**, 125327 (2009).
[7] Mounet, N. *et al.*, "Two-dimensional materials from high-throughput computational exfoliation of experimentally known compounds," Nat. nanotechnol. **13**, 246–252 (2018).
